# Competencies of hospital managers – A systematic scoping review

**DOI:** 10.3389/fpubh.2023.1130136

**Published:** 2023-03-23

**Authors:** Costase Ndayishimiye, Katarzyna Dubas-Jakóbczyk, Anastasia Holubenko, Alicja Domagała

**Affiliations:** ^1^Department of Health Economics and Social Security, Institute of Public Health, Jagiellonian University Medical College, Kraków, Poland; ^2^Faculty of Medicine, Jagiellonian University Medical College, Kraków, Poland; ^3^Department of Health Policy and Management, Institute of Public Health, Jagiellonian University Medical College, Kraków, Poland

**Keywords:** hospital manager, competencies, skills, hospital administration, hospital management

## Abstract

Hospital managers around the world work under constant pressure to adapt their organizations to new challenges and health policy goals. This requires a comprehensive set of competencies. The objective of this scoping review was to identify, map, and systematize the literature on hospital manager competencies. The review involved six steps: (1) defining research questions; (2) identifying relevant literature; (3) selecting publications; (4) data extraction; (5) data analysis and result reporting; and (6) consultations. A total of 57 full-text publications were included (46 empirical studies, six literature reviews, four expert opinions/guidelines, and one dissertation). Interest in this topic has grown in recent years, with most of the identified studies published since 2015. The empirical studies fall into three major groups: 34.8% (16/46) examined hospital managers’ competencies in terms of their types or classifications; 30.4% (14/46) focused on their measurement; and 30.4% (14/46) examined both aspects. In majority of studies, both ‘hard competencies,’ such as specific technical knowledge or skills acquired through practical training, and ‘soft competencies,’ e.g., adaptability, leadership, communication, teamwork, are echoed for effective hospital management. These point out the importance of both ‘external’ formal education trainings as well as ‘internal’ peer-support and/or coaching as complementary competency improvement approaches. This scoping review helps build a knowledge base around the topic and provides implications for future research. The latter can involve: a targeted systematic review addressing the methods for measuring the level of competence of hospital managers or studies focused on identifying the need for new types of competencies.

## 1. Introduction

Healthcare systems around the world are constantly facing multiple challenges, including the growing healthcare needs of an aging population, shortages of healthcare professionals, and the rising cost of healthcare services. In recent years in particular, the pressure on healthcare systems has increased tremendously. The COVID-19 pandemic has accelerated and forced further rapid change, as well as the need for resilient adjustments at both the system and organizational levels (1, 2). Hospitals are unique and complex types of organizations that constitute the core of health care providers. They function in a highly regulated environment and operate under pressure to adjust to changes in both health policy objectives and external determinants (2, 3). These challenges influence the demand for developing and/or enhancing hospital managers’ competencies, which are critical to effectively manage and improve hospital productivity. Currently, healthcare managers are operating in a vastly changing hospital environment, including tremendous workforce shortages (4), changing models of care (4, 5), pressures related to cybersecurity issues (6), and the “green hospital” movement (7). Hence, the need to examine and develop the competencies with which they should be equipped to address recent realities in hospital management. The competency-based approach is based on the level of knowledge, skills, and abilities of employees (8). The evidence suggests the existence of a core management competencies model for hospital managers (9–12).

Guided by the general objectives of scoping reviews, we aimed at identifying, mapping, and systemizing the literature on hospital manager competencies (13, 14). Our objective was to review the existing studied by classifying identified items by a pre-defined set of criteria (e.g., when, where and what type of studies were published; what was their main focus; what methods were applied, etc.).We aimed at exploring the breadth of existing literature (15), including empirical research, technical reports, expert opinions, dissertations, etc., and identifying potential research gaps. Several thematically related literature reviews have already been published. However, these either focused on specific types of competencies and/or included only certain types of empirical research (12, 16, 17) or were narrowed to a limited number of countries (18). As such, our aim was to provide a comprehensive overview and to map the existing literature into three predefined categories: (1) type/classification of competencies (were existing competency models used?); (2) their measurement (what instruments were used?); and (3) the relationship between hospital managers’ competencies and hospital-level outcomes (what metrics were used?).

## 2. Methods

A systematic scoping literature review was conducted following the methodological guidelines developed by Arksey and O’Malley (19) and updated by Levac et al. (20). The work involved six sequential stages: (1) definition of specific research questions; (2) identification of relevant literature; (3) selection of evidence; (4) extraction of evidence; (5) data analysis, summary and reporting of findings; and (6) consultations. The review protocol was registered with the Open Science Framework (21). Results were reported using the PRISMA-ScR checklist (22).

### 2.1. Defining review questions

The following specific review questions (RQ) have been defined:

1. What periods and geographical areas are covered by the identified publications?
2. What type of publications are available?
3. What is the focus of the identified publications?
4. What methods were used in the identified empirical studies?
5. What results were obtained or what conclusions were drawn?

### 2.2. Identification of the relevant literature

We searched the following seven databases: (1) Medline *via* PubMed; (2) Web of Science; (3) Scopus; (4) ABI/INFORM *via* ProQuest; (5) APA PsyclInfo *via* EBSCO; (6) CINAHL *via* EBSCO; (7) Business Source Complete *via* EBSCO. The search strategy was developed iteratively. We combined terms from two core themes: (1) hospital manager AND (2) competency. Multiple synonyms were used for each theme (Supplementary Table S1). We searched for terms in the title and/or abstract published since 2000. The search was conducted in June 2022. The reference lists of publications included in the review were manually searched to find additional studies of interest. In addition, websites of international organizations active in the field of health management and scientific journals on this topic were also manually searched (Supplementary Table S2).

### 2.3. Selection of publications

Publications were selected in two steps: screening of abstracts and assessment of full texts based on predefined inclusion and exclusion criteria. Studies were included (1) if they dealt with the competencies of senior and middle-level hospital managers; (2) if they were peer-reviewed empirical studies, theoretical papers, technical reports, expert opinions/guidelines books, chapters or dissertations; and (3) if the full text was in English. They were excluded if: (1) they did not focus on the competencies of middle- or senior level hospital mangers (this exclusion covered, among others, three main subcategories: studies on first-line hospital management physicians/nurses practice managers/coordinators, clinical team managers/leaders, defined in our review as those who have having direct contact with patients; studies on managers working outside the hospital settings – e.g. in primary, community, or long-term care; studies examining the knowledge or attitudes of hospital managers, but in some narrowly defined areas such as, waste management and infection control); (2) had the incorrect type of publication (book reviews, commentaries, cover letters, conference abstracts, or other types publications that are not full texts); (3) full texts in were in another language. The justification behind decision to exclude studies on mangers involved directly with patent care – e.g. case managers (defined by us as first line managers) was based both on the review’s focus on hospital managers with the greatest influence/decision making power on the organization as a whole and on the pragmatic approach of not mixing organizational management competencies with those related to direct patient care, e.g., within coordinated care programs. The decision to exclude studies focusing on hospital managers’ knowledge in some narrowly defined topic, like waste management was also based on our review more comprehensive approach to competencies.

Two researchers (CN and AH) participated in both phases in parallel (to compare the selection and assess the level of agreement). In case of disagreement, a third researcher (KDJ) was involved in the process, to discuss and reach a final agreement. The designated software was used (Mendeley and Rayyan).

### 2.4. Data extraction

Data extraction tables were developed using MS Excel forms and tailored to the type of publications included (separate tables were used for empirical studies and other types of publications). Each section of the extraction table was matched with the specific research question. The extraction was also conducted independently by two researchers (CN and AH), then compared and finalized. Supplementary Table S3 provides an example of the data extraction table for empirical studies.

### 2.5. Data analysis and reporting

Both quantitative and qualitative methods (thematic analysis) were used for data analysis and synthesis. The included publications were grouped according to predefined categories. The codes assigned in the extraction table were used for quantitative summaries. For example, based on the main focus of empirical studies, three categories were distinguished: (1) studies on the types or classifications of hospital managers’ competencies; (2) measurement of competencies; and (3) the relationship between competencies and hospital-level outcomes. The results of the review were compiled in the form of tables.

### 2.6. Consultations

The preliminary findings were presented at the internal seminar at the authors’ institution in December 2022, attended by 40 hospital managers and healthcare management experts. The purpose of the consultation was to validate the results and provide directions for further, in-depth research.

## 3. Results

### 3.1. Search results

Searches in seven databases yielded 9,764 records (Supplementary Tables S4–S10 show the search results for each database). A further 22 records were identified through manual searches of relevant organizations and journal websites. After removing duplicates, 4,378 records were screened for titles and abstracts, after which 189 publications were retrieved for full-text analysis. A total of 42 publications met the inclusion criteria. Screening of the reference list led to the inclusion of 15 additional articles. Thus, 57 publications were included in the final synthesis (9, 10, 12, 16, 18, 23–74). A PRISMA flow chart of the results is attached (Supplementary Figure S1). Supplementary Table S11 provides a list of all included studies by year, country, and type of publication, as well as the full reference information.

### 3.2. Type of publications

**Figure 1:**
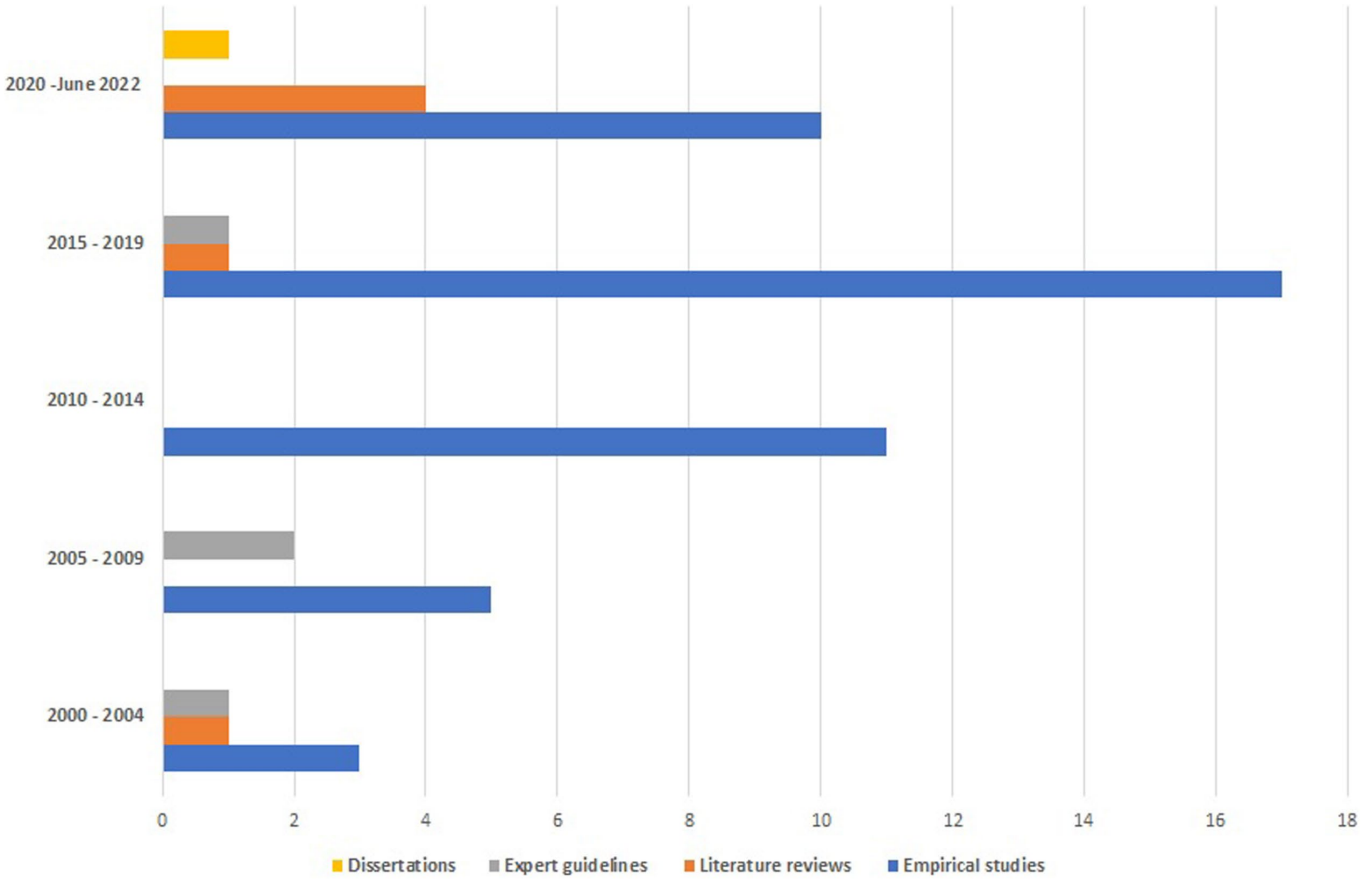
Number of items included per 5-year publication period.

The 57 publications include 46 empirical studies (10, 23–63, 71–74), six literature reviews (12, 16, 18, 64–66), four publications classified as expert opinions/guidelines (9, 67–69) and one dissertation (70). The publications were published between 2002 and 2022, with the majority (60% or 34/57) published since 2015 (Figure 1).

Five of the six included literature reviews have been published since 2015. Two reviews (64, 66) did not report the number of included studies, while the others included between 12 and 33 studies. The review of various aspects of competencies of senior and middle-levels healthcare managers was the focus of all six reviews (Supplementary Table S12). Three reviews investigated the types or classifications of competencies required by hospital managers in specific thematic areas {e.g., leadership (12), value-based care (16) or general competencies in a small number of specific countries [e.g., developing countries (18)]}. One review provided a rather broad overview of issues related to *competency-based education and training* for health care managers (64). The remaining two reviews (65, 66) analyzed healthcare managers’ leadership competencies. The four expert guidelines provided recommendations on general competencies for hospital management, all focused on United States (US) health system (9, 67–69). Finally, the PhD thesis (70) was also from the US and analyzed the scope of managerial competencies that drive successful change initiatives using several empirical examples.

### 3.3. Empirical studies classification

Table 1 shows the general overview of 46 empirical studies. Geographically, most studies were from Asian countries (36% or 16/ 46), followed by the Americas (*n* = 10), Australia (*n* = 9), Africa (*n* = 6), and Europe (*n* = 4) (Supplementary Figure S2). One study included countries from two different geographical regions (i.e., different continents), namely Brazil and Australia (44). When broken down by country, 57% (*n* = 26/46) of all studies were from only three countries, namely Iran (*n* = 10), Australia (*n* = 9), and the United States (*n* = 7).

**Table 1:** Empirical studies overview.

Ref. no	Abbrev.	Country	Study main topic^a^	Type of managers^b^	Type of study^c^
(63)	Abdi et al. (2022)	Iran	T	Not stated	QL
(74)	Barati et al. (2016)	Iran	T	S	QL
(23)	Collins et al. 2015	US	T	S	QN
(24)	Dadgar et al. (2012)	Iran	T	M	QL
(10)	Garman et al. (2011)	US	T	S, M	QN
(30)	Kakemam et al. (2021)	Iran	T	S, M	QL
(34)	Leggat (2007)	Australia	T	S	QN
(35)	Lehr et al. (2011)	Germany	T	S	QN
(37)	Liang et al. (2010)	Australia	T	S	Mix
(39)	Liang et al. (2018)	Australia	T	S, M	Mix
(43)	Mahdavi et al.(2020)	Iran	T	M, S	QL
(50)	Pillay (2008)	South Africa	T	S, M	QN
(55)	Shewchuk et al. (2005)	US	T	S	Mix
(58)	Van Tuong and Thanh (2017)	Vietnam	T	M, S	Mix
(62)	Wallick (2002)	US	T	S, M	QL
(59)	Wongprasit (2014)	Thailand	T	S	QL
(73)	Babinski (2016)	US	T, M	S, M, N	QN
(26)	Guo (2003)	US	T, M	S	QL
(61)	Howard et al. (2018)	Australia	T, M	S, M	QN
(28)	Jafari et al. (2019)	Iran	T, M	S, M, N	QN
(32)	Khadka et al. (2014)	Nepal	T, M	M, S	QN
(40)	Liang et al. (2013)	Australia	T, M	M, S	mix
(41)	Liang et al. (2020)	China	T, M	S	mix
(42)	MacKinnon et al. (2004)	Canada	T, M	S, M, N	QN
(44)	Martins et al. (2022)	Australia, Brazil	T, M	S	mix
(46)	Messum et al. (2016)	Australia	T, M	M	QN
(51)	Pillay (2008)	South Africa	T, M	S	QN
(53)	Pillay (2010)	South Africa	T, M	S	QN
(54)	Ramirez et al. (2019)	Mexico	T, M	S, M	QN
(56)	Shojaei et al. (2011)	Iran	T, M	S, M	QN
(57)	Toygar and Akbulut (2013)	Turkey	T, A	S, M, N	Mix
(71)	Aini (2018)	Indonesia	M	S, M	Mix
(25)	Fanelli et al. (2021)	Italy	M	M	QN
(29)	Kakemam and Dargahi (2019)	Iran	M	S	QN
(31)	Kalhor et al. (2016)	Iran	M	S	QN
(33)	Landry et al. (2012)	US	M	S, M	QN
(36)	Liang et al. (2016)	Australia	M	M	QN
(38)	Liang et al. (2017)	Australia	M	M	QN
(27)	Liang et al. (2018)	Australia	M	M	QN
(60)	Lockhart and Backman (2009)	Canada	M	S, M	mix
(45)	Mehrnoosh et al. (2020)	Iran	M	S, M, N	QN
(47)	Ogbonnia et al. (2018)	Nigeria	M	S, M	QN
(48)	Okonkwo et al. (2020)	Nigeria	M	M	QN
(49)	Patnaik et al. (2017)	India	M	S	QN
(52)	Pillay (2008)	South Africa	M	S, M	QN
(72)	Aktan and Sahin (2021)	Turkey	A	S, M	QN

^a^T, type; classification; M, measurement; A, associations; O, other. ^b^S, senior; M, middle; N, nursing. ^c^QL, qualitative; QN, quantitative, mix.

Of the 46 empirical studies (shown in Table 1), 34.8% (16/46) contained information on the types/classifications of hospital managers’ competencies, 30.4% (14/46) included information on their measurement, 30.4% (14/46) included both of these two aspects, and one study examined both types of competencies and their relationship simultaneously. The latter identified the levels of decision-making and problem-solving skills of hospital administrators and determined the interrelationships between these skills and other administrative skills (57). Only one empirical study focused exclusively on examining the relationships between hospital managers’ characteristics (including competencies) and resource management capacity (72). Most studies (38/46) focused on the competencies of senior-level hospital managers, including 23 that analyzed the competencies of senior and middle-level managers simultaneously.

Of the 45 studies that focused on the types and/or measurement of hospital managers’ competencies, 60% (27/45) used quantitative methods (mostly surveys and questionnaires, followed by statistical analyses), 17.8% (8/45) used qualitative methods (interviews with thematic analysis), and 22.2% (10/45) used a mixed-methods approach. Majority of studies reported that both ‘hard competencies’ such as specific technical knowledge (44, 48) or skills acquired through practical training (41, 44, 63, 72), and ‘soft competencies,’ e.g., adaptability (63), leadership (30, 43, 48, 67, 69), effective communication (9, 30, 44, 45, 48, 67, 69), teamwork (9, 30, 44), time management (43–45, 72), creativity (43, 44, 48), and decision-making (43, 67), are needed for effective hospital management. As a consequence, many researchers pointed out the importance of both ‘external’ formal education training/certificates as well as ‘internal’ peer-support, mentoring, and/or coaching as complementary competency improvement approaches.

The majority of studies that focused on measuring the level of hospital managers’ competencies applied a quantitative approach (79%, or 22/28). The researchers either developed their own questionnaire/survey for the purpose of their study (*n* = 15/28) or used existing tools (*n* = 10/28). Examples of existing, validated tools included the Management Competence Assessment Tool for Health Care Managers (MCAP) used in three studies (38, 39, 41), and the questionnaire designed by the International Hospital Federation (IHF) used in two studies (28, 54). The first includes elements related to professionalism and is divided into six core competencies: (1) evidence (evidence-based decision-making); (2) resources (operation, administration, and management of resources); (3) knowledge (knowledge of organizational health environments and communications); (4) interpersonal communication skills and relationship management; (5) leadership (leadership of people and organizations); and 6. change (enable and manage changes). The IHF questionnaire consists of 80 competencies contained within five domains, namely: (1) business, (2) communication and relationship management, (3) health and healthcare environment, (4) leadership, and (5) professional and social responsibility. In all studies, the measurement of the level of competencies was “subjective” – hospital managers themselves rated their competencies. An additional ‘cross – check’ component was added in five studies (36, 38, 39, 56, 61). The latter included either an additional survey conducted among supervisors (61) or a 360-degree feedback process (36, 38, 39, 56). In the latter case, hospital managers’ competencies were also assessed by their peers [e.g., community managers (36)].

## 4. Discussion

Our review identified and systematized the literature on hospital manager competencies. Between 2000 and June 2022, a total of 57 full text English publications were published, including 46 empirical studies (10, 23–63, 71–74), six literature reviews (12, 16, 18, 64–66), four expert opinions/guidelines (9, 67–69) and one doctoral dissertation (70). The review showed increasing interest in the topic, with most studies released since 2015. Most of the 46 empirical studies were from Asian countries (36% or 16/46), followed by the Americas (*n* = 10) and Australia (*n* = 9). Also, all expert opinions/guidelines were from the United States. The geographical distribution of the identified publications indicates a research gap in both theoretical/conceptual and empirical research on hospital manager competencies in European settings. This finding is partly in line with the results of other studies showing that there is a lack of research on hospital financial management in Europe compared to the US (75). This may be related to the traditionally less market oriented health system structures in European countries and the slower adaptation of most up-to-date management solutions.

The empirical studies focused primarily on the competencies of senior-level hospital managers and included an analysis of their types or classification (definition of the main dimensions of competencies) or measurement (subjective assessment of the level of competency). The review found a research gap in terms of empirical studies focusing on the relationship between hospital managers competencies and hospital-level outcomes. However, the latter, may be the result of our inclusion criteria for the review. As we excluded studies that focus on hospital managers’ knowledge in some specific, narrowly defined topic we have also excluded studied that could, for example, assess the association between hospital mangers’ knowledge in financial management and hospital financial metrics outcomes.

Studies on hospital managers’ competencies are related to their main tasks and challenges. Currently, hospital managers are focused on many pressing priorities, including the expansion of care delivery, significant staff shortages, the rapid development of new medical technologies, and the use of Big Data. Based on the results of the study conducted in 2022 among 3000 managers from 15 countries, the three core priorities for hospital leaders have been indicated as follows: (1) improving the staff experience (including job satisfaction and retention); (2) bridging the gap between the promise of predictive analytics and current usage (as an opportunity to improve the quality of care, reducing costs, and speed healthcare delivery); (3) addressing threats to healthcare data security (76). Such fundamental changes require urgent shift in managers’ priorities, a reassessment of their training needs, and the acquisition of a range of new competencies. Hospital managers and experts who participated in our consultations also emphasized the need for a stronger focus on soft skills. The development of these capabilities is necessary for effective human resources management (including staff motivation, protection of employees’ well-being and mental health, team building, and management of interprofessional and age-diverse team management) and successful collaboration with different stakeholders. These recommendations are in line with the latest World Health Organization report on health workforce (4).

The main strengths of our review compared to previously published reviews, e.g., Kakemam et al. (12) or Walsch et al. (16), are: a broader scope of sources searched (seven databases plus a screening relevant organizational websites and journals); broader inclusion criteria (e.g., inclusion of grey literature; inclusion of studies on the association between competencies and hospital outcomes); and provisions of geographical classification of the published literature. The main limitations are that only English-language publications were included and that the quality of the included studies was not assessed. While the latter is in line with the guidelines for conducting scoping literature reviews (13), it limits our ability to formulate implications for policy makers. In addition as mentioned earlier, for the feasibility of the study, this review excluded studies that focused on a narrowly defined scope of hospital managers knowledge.

Despite these limitations, our scoping review contributes to building a knowledge base around the topic of hospital managers’ competencies and provides implications for further research. For example, a future, targeted systematic review could address the methods used to measure hospital managers’ competency levels. Such a systematic review could ask a narrow research question about the use of 360-degree feedback to evaluate competencies. This model provides an objective, multi-sourced and comprehensive assessment (77) and its application in healthcare setting is growing (78). More evidence on how to adequately measure hospital managers’ competencies is a prerequisite for planning research on the impact of these competencies. New, appropriately planned original research could focus on identifying the needs for upgraded competencies that arise from the current problems and challenges facing health systems. The latter could include emergency and disaster risk management (79), cyber security (6), and changes in health workforce structures (4, 5). Finally, the deficits in theoretical and empirical work on hospital managers’ competencies in the European context represent an important research gap that deserves further investigation.

## Author contributions

KD-J: conceptualization and supervision. KD-J and AD: methodology. KD-J, CN, and AH: acquisition of data and data analysis. KD-J, CN, and AD: findings interpretation and manuscript writing. KD-J, AD, and CN: manuscript review and revision. All authors meet the authorship criteria and agree to the submission of the manuscript, made substantial contributions to the conception or design of the work, according to the International Committee of Medical Journal Editors (ICMJE) and to the Committee on Publication Ethics (COPE).

## Funding

This publication arises from the project funded by National Science Centre Poland (No 2022/06/X/HS4/00040) entitled: Hospital managers competencies – a systematic scoping review.

## Conflict of interest

The authors declare that the research was conducted in the absence of any commercial or financial relationships that could be construed as a potential conflict of interest.

## Publisher’s note

All claims expressed in this article are solely those of the authors and do not necessarily represent those of their affiliated organizations, or those of the publisher, the editors and the reviewers. Any product that may be evaluated in this article, or claim that may be made by its manufacturer, is not guaranteed or endorsed by the publisher.
