# Supplementary material for: Competencies of hospital managers – A systematic scoping review
Source: Front Public Health. 2023 Mar 23;11:1130136. doi: 10.3389/fpubh.2023.1130136 (PMC10076734; doi:10.3389/fpubh.2023.1130136)

Supplementary Material

Competencies of hospital managers - a systematic scoping review

Costase Ndayishimiye, Katarzyna Dubas-Jakóbczyk, Anastasia Holubenko, Alicja Domagała

* Correspondance: Katarzyna Dubas-Jakóbczyk: Katarzyna.Dubas@uj.edu.pl

SUPPLEMENTARY TABLES

Supplementary Table S1. Search terms for the databases.

Topic	Search terms
**hospital manager**	"hospital manager*" OR "hospital management" OR "hospital director*" OR "hospital administrator*" OR "hospital president*" OR "hospital leader*" OR "hospital executive*" OR "health care manager*" OR "healthcare manager*" OR "health services manager*" OR "healthcare administrator*" OR "health services administrator*" OR "health care administrator*" OR "healthcare director*" OR "health services director*"
**competencies**	competenc* OR skill* OR abilit* OR attitude* OR knowledge OR qualification* OR capacit* OR capabilit* OR expertise

Supplementary Table S2. List of hand-searched organizations and journals, their websites and search dates.

№	Organization/Journal	Website	Search Date
1	The European Association of Hospital Managers (EAHM)	https://eahm.eu.org/	30.06.2022
2	The European Hospital and Healthcare Federation (HOPE)	https://hope.be/	30.06.2022
3	The European Health Management Association (EHMA)	https://ehma.org/	30.06.2022
4	American Hospital Association (AHA)	https://www.aha.org/	30.06.2022
5	Journal of Healthcare Leadership	https://www.dovepress.com/journal-of-healthcare-leadership-journal	30.06.2022
6	The Health Care Manager *(*HCM*)* Journal	https://journals.lww.com/healthcaremanagerjournal/pages/default.aspx	30.06.2022
7	International Hospital Federation (IHF)	https://www.ihf-fih.org/	01.07.2022
8	Journal of Health Organization and Management/Health Care Management Review	https://www.dovepress.com/journal-of-healthcare-leadership-journal	01.07.2022
9	Health Services Management Research.	https://journals.sagepub.com/home/hsm	02.07.2022

10	Australasian College of Health Service Management.	https://www.achsm.org.au/	06.07.2022
11	Institute of Health & Social Care Management	https://ihm.org.uk/about-us/	10.07.2022
12	The American Association of Healthcare Administrative Management (AAHAM)	https://www.aaham.org/	10.07.2022
13	American College of Healthcare Executives (ACHE)	https://www.ache.org/	10.07.2022
14	American Health Information Management Association (AHIMA)	https://www.ahima.org/	10.07.2022
15	The Healthcare Financial Management Association (HFMA)	https://www.hfma.org/	10.07.2022
16	Medical Group Management Association (MGMA**)**	https://www.mgma.com/	10.07.2022

Supplementary Table S3. Data Extraction Form.

**Research question**	**Data to be extracted**	**Coding examples**
RQ1	authors/title	N/A
	year of publication	• 2000 –2005<br>• 2006 – 2010<br>• 2011-2015<br>• after 2015
	research country	N/A (list of countries)
	language of the publication	• English
RQ2	type of publication	• empirical study<br>○ secondary data (review) vs primary data (quantitative vs qualitative vs mixed methods)<br>• theoretical paper<br>• technical report<br>• formal guidelines
RQ3	focus of publication	• type/classification of competencies<br>• measurement of competencies<br>• association between competencies and hospital outcomes
RQ4	Methods applied	• quantitative (e.g., questionnaire)<br>• qualitative (e.g., interviews, Delphi-study)<br>• mixed
RQ5	Results/conclusions	• results achieved/conclusions stated

Supplementary Tables S4-S10. Search results per database:

Database 1: ABI/INFORM
Date of the search: 17/06/2022

**Query**	**Results**
ab("hospital manager*" OR "hospital management" OR "hospital director*" OR "hospital administrator*" OR "hospital president*" OR "hospital leader*" OR "hospital executive*" OR "health care manager*" OR "healthcare manager*" OR "health services manager*" OR	985

"healthcare administrator*" OR "health services administrator*" OR "health care administrator*" OR "healthcare director*" OR "health services director*") AND ab(competence* OR skill* OR abilit* OR attitude* OR knowledge OR qualification* OR capacit* OR capabilit* OR expertise)	

Database 2: Business Source Complete
Date of the search: 20/06/2022

Query	Results
AB (“hospital manager*” OR “hospital management” OR “hospital director*” OR “hospital administrator*” OR “hospital president*” OR “hospital leader*” OR “hospital executive*” OR “health care manager*” OR “healthcare manager*” OR “health services manager*” OR “healthcare administrator*” OR “health services administrator*” OR “health care administrator*” OR “healthcare director*” OR “health services director*”) AND AB (competence* OR skill* OR abilit* OR attitude* OR knowledge OR qualification* ...	494
AB (“hospital manager*” OR “hospital management” OR “hospital director*” OR “hospital administrator*” OR “hospital president*” OR “hospital leader*” OR “hospital executive*” OR “health care manager*” OR “healthcare manager*” OR “health services manager*” OR “healthcare administrator*” OR “health services administrator*” OR “health care administrator*” OR “healthcare director*” OR “health services director*”) AND AB (competence* OR skill* OR abilit* OR attitude* OR knowledge OR qualification* ...	511
AB (“hospital manager*” OR “hospital management” OR “hospital director*” OR “hospital administrator*” OR “hospital president*” OR “hospital leader*” OR “hospital executive*” OR “health care manager*” OR “healthcare manager*” OR “health services manager*” OR “healthcare administrator*” OR “health services administrator*” OR “health care administrator*” OR “healthcare director*” OR “health services director*”) AND AB (competence* OR skill* OR abilit* OR attitude* OR knowledge OR qualification* ...	593

Database 3: CINAHL Complete
Date of the search: 17/06/2022

Query	Results
AB ("hospital manager*" OR "hospital management" OR "hospital director*" OR "hospital administrator*" OR "hospital president*" OR "hospital leader*" OR "hospital executive*" OR "health care manager*" OR "healthcare manager*" OR "health services manager*" OR "healthcare administrator*" OR "health services administrator*" OR "health care administrator*" OR "healthcare director*" OR "health services director*") AND AB (competence* OR skill* OR abilit* OR attitude* OR knowledge OR qualification* OR capacit* OR capabilit* OR expertise)	1,085

Database 4: PsycInfo
Date of the search: 20/06/2022

Query	Results
AB (“hospital manager*” OR “hospital management” OR “hospital director*” OR “hospital administrator*” OR “hospital president*” OR “hospital leader*” OR “hospital executive*” OR “health care manager*” OR “healthcare manager*” OR “health services manager*” OR “healthcare administrator*” OR “health services administrator*” OR “health care administrator*” OR “healthcare director*” OR “health services director*”) AND AB (competence* OR skill* OR abilit* OR attitude* OR knowledge OR qualification* ..	502
AB (“hospital manager*” OR “hospital management” OR “hospital director*” OR “hospital administrator*” OR “hospital president*” OR “hospital leader*” OR “hospital executive*” OR “health care manager*” OR “healthcare manager*” OR “health services manager*” OR “healthcare administrator*” OR “health services administrator*” OR “health care administrator*” OR “healthcare director*” OR “health services director*”) AND AB (competence* OR skill* OR abilit* OR attitude* OR knowledge OR qualification* ...	

Database 5: PubMed
Date of the search: 17/06/2022

Query	Results
("hospital manager*"[Title/Abstract] OR "hospital management"[Title/Abstract] OR "hospital director*"[Title/Abstract] OR "hospital administrator*"[Title/Abstract] OR "hospital president*"[Title/Abstract] OR "hospital leader*"[Title/Abstract] OR "hospital executive*"[Title/Abstract] OR "health care manager*"[Title/Abstract] OR "healthcare manager*"[Title/Abstract] OR "health services manager*"[Title/Abstract] OR "healthcare administrator*"[Title/Abstract] OR "health services administrator*"[Title/Abstract] OR "health care administrator*"[Title/Abstract] OR "healthcare director*"[Title/Abstract] OR "health services director*"[Title/Abstract]) AND (competence*[Title/Abstract] OR skill*[Title/Abstract] OR abilit*[Title/Abstract] OR attitude*[Title/Abstract] OR knowledge[Title/Abstract] OR qualification*[Title/Abstract] OR capacit*[Title/Abstract] OR capabilit*[Title/Abstract] OR expertise[Title/Abstract]) Filters: Full text, English, from 2000 – 2022	1,900

Database 6: Scopus
Date of the search: 18/06/2022

Query	Results
("hospital manager*" OR "hospital management" OR "hospital director*" OR "hospital administrator*" OR "hospital president*" OR "hospital leader*" OR "hospital executive*" OR "health care manager*" OR "healthcare manager*" OR "health services manager*" OR "healthcare administrator*" OR "health services administrator*" OR "health care administrator*" OR "healthcare director*" OR "health services director*") AND TITLE-ABS (competence* OR skill* OR abilit* OR attitude* OR knowledge OR qualification* OR capacit* OR capabilit* OR expertise) AND (LIMIT-TO (PUBYEAR , 2022) OR LIMIT-TO (PUBYEAR , 2021) OR LIMIT-TO (PUBYEAR , 2020) OR LIMIT-TO (PUBYEAR , 2019) OR LIMIT-TO (PUBYEAR , 2018) OR LIMIT-TO (PUBYEAR , 2017) OR LIMIT-TO (PUBYEAR , 2016) OR LIMIT-TO (PUBYEAR , 2015) OR LIMIT-TO (PUBYEAR , 2014) OR LIMIT-TO (PUBYEAR , 2013) OR LIMIT-TO (PUBYEAR , 2012) OR LIMIT-TO (PUBYEAR , 2011) OR LIMIT-TO (PUBYEAR , 2010) OR LIMIT-TO (PUBYEAR , 2009) OR LIMIT-TO (PUBYEAR , 2008) OR LIMIT-TO (PUBYEAR , 2007) OR LIMIT-TO (PUBYEAR , 2006) OR LIMIT-TO (PUBYEAR , 2005) OR LIMIT-TO (PUBYEAR , 2004) OR LIMIT-TO (PUBYEAR , 2003) OR LIMIT-TO (PUBYEAR , 2002) OR LIMIT-TO (PUBYEAR , 2001) OR LIMIT-TO (PUBYEAR , 2000)) AND (LIMIT-TO (DOCTYPE , "ar") OR LIMIT-TO (DOCTYPE , "re") OR LIMIT-TO (DOCTYPE , "ch") OR LIMIT-TO (DOCTYPE , "bk")) AND (LIMIT-TO (LANGUAGE , "English"))	2,825

Database 7: Web of Science
Date of the search: 17/06/2022

Query	Results

6	**#2 AND #1** and **2000** or **2001** or **2002** or **2003** or **2004** or **2005** or **2006** or **2007** or **2008** or **2009** or **2011** or **2012** or **2013** or **2014** or **2015** or **2016** or **2017** or **2018** or **2019** or **2020** or **2021** or **2022** (Publication Years) and **Articles** or **Review Articles** or **Book Chapters** (Document Types) and **English** (Languages)	1,973	1,973
5	**#2 AND #1** and **2000** or **2001** or **2002** or **2003** or **2004** or **2005** or **2006** or **2007** or **2008** or **2009** or **2011** or **2012** or **2013** or **2014** or **2015** or **2016** or **2017** or **2018** or **2019** or **2020** or **2021** or **2022** (Publication Years) and **Articles** or **Review Articles** or **Book Chapters** (Document Types)	2,083	
4	**#2 AND #1** and **2000** or **2001** or **2002** or **2003** or **2004** or **2005** or **2006** or **2007** or **2008** or **2009** or **2011** or **2012** or **2013** or **2014** or **2015** or **2016** or **2017** or **2018** or **2019** or **2020** or **2021** or **2022** (Publication Years)	2,255	
3	**#2 AND #1**	2,440	
2	**(TI=(competence* OR skill* OR abilit* OR attitude* OR knowledge OR qualification* OR capacit* OR capabilit* OR expertise)) OR AB=(competence* OR skill* OR abilit* OR attitude* OR knowledge OR qualification* OR capacit* OR capabilit* OR expertise)**	6,248,791	
1	**(TI=("hospital manager*" OR "hospital management" OR "hospital director*" OR "hospital administrator*" OR "hospital president*" OR "hospital leader*" OR "hospital executive*" OR "health care manager*" OR "healthcare manager*" OR "health services manager*" OR "healthcare administrator*" OR "health services administrator*" OR "health care administrator*" OR "healthcare director*" OR** [illegible]	9,356	

Supplementary Table S11. A list of the included 57 studies–full reference, publication year, country & study type.

No	Full reference	Year of Publication	Country	Publication type
**1**	Abdi, Z. et al. (2022) ‘Role of hospital leadership in combating the COVID-19 pandemic.’, Health services management research, 35(1), pp. 2–6. doi: 10.1177/09514848211035620.	2022	Iran	empirical
**2**	Aini, Q. (2018) ‘Management skill and leadership: A case study from Hospital managers of charity business in health’, Journal of Social Sciences Research, 4(12), pp. 478–482. doi: 10.32861/jssr.412.478.482.	2018	Indonesia	empirical
**3**	Aktan, T., & Sahin, B. (2021). Assessment of Relationship between Hospital Resource Management Capacity and Characteristics of Hospitals and Managers. KONURALP TIP DERGISI, 13(2), 299–311. https://doi.org/10.18521/ktd.912698	2021	Turkey	empirical
**4**	Anderson P., Pulich M. (2002). Managerial Competencies Necessary in Today's Dynamic Health Care Environment. The Health Care Manager *(*HCM*)* Journal, 21(2), 1-11.	2002	US	other - Guidelines
**5**	Babinski P. J. (2016). The Perceived Importance of Role-Specific Competencies for Health Care Leaders Establishes the Need to Expand Role Theory. The Health Care Manager (HCM) Journal, 35(2), 164-179. https://journals.lww.com/healthcaremanagerjour-nal/Abstract/2016/04000/The_Perceived_Importance_of_Role_Specific.11.aspx	2016	US	empirical
**6**	Barati, O., Sadeghi, A., Khammarnia, M., Siavashi, E., & Oskrochi, G. (2016). A Qualitative Study to Identify Skills and Competency Required for Hospital Managers. Electronic Physician, 8(6), 2458–2465. https://doi.org/10.19082/2458	2016	Iran	empirical
**7**	Calhoun, J.G., Dollett, L., Sinioris, M.E., Wainio, J.A., Butler, P.W., Griffith, J.R. and Warden, G.L. (2008), “Development of an interprofessional competency model for healthcare leadership”, Journal of Healthcare Management, Vol. 53 No. 6, pp. 375-391. https://journals.lww.com/jhmonline/Citation/2008/11000/Development_of_an_Interprofessional_Competency.6.aspx	2008	US	other - Guidelines
**8**	Calhoun, J. G., Davidson, P. L., Sinioris, M. E., Vincent, E. T., & Griffith, J. R. (2002). Toward an understanding of competency identification and assessment in health care management. Quality Management in Healthcare, 11(1), 14–38.	2002	N/A	other – Review article
**9**	Collins S. K., Collins K. S. (2015). Leadership Characteristics for Health Care Managers: Perspectives of Chief Executive Officers in US Hospitals. The Health Care Manager (HCM) Journal, 34(4), 293-296. https://journals.lww.com/healthcaremanagerjour-nal/Abstract/2015/10000/Leadership_Characteristics_for_Health_Care.4.aspx	2015	US	empirical
**10**	Dadgar, E., Janati, A., Tabrizi, J. S., Asghari-Jafarabadi, M., & Barati, O. (2012). Iranian Expert Opinion about Necessary Criteria for Hospitals Management Performance Assessments. Health Promotion Perspectives, 2(2), 223–230. https://doi.org/10.5681/hpp.2012.027	2012	Iran	empirical
**11**	Fanelli S., Pratici L., Zangrandi A. (2021). Managing healthcare services: Are professionals ready to play the role of manager? Health Services Management Research, 0(0), 1-11. https://journals.sagepub.com/doi/full/10.1177/09514848211010264	2021	Italy	empirical

**12**	Flaig, J., Alam, A., Huynh, J., Reid-Hector, J., & Heuer, A. (2020). Examining How Formal Leadership Development Programs Positively Influence Hospital Leaders' Individual Competencies and Organizational Outcomes - An Evidence-Based Literature Review. *Journal of Healthcare Leadership*, *12*, 69–83. https://doi.org/10.2147/JHL.S239676	2020	N/A	other – Lit. review
**13**	Freed, D. H. (2017). Ten Core Competencies for Hospital Administrators. *Health Care Manager*, *36*(2), 108–115. https://doi.org/10.1097/HCM.0000000000000154	2017	US	other - Guidelines
**14**	Garman AN, Scribner L. Leading for quality in healthcare: development and validation of a competency model. J Healthc Manage. 2011;56(6):373-384. https://journals.lww.com/jhmonline/Abstract/2011/11000/Leading_for_Quality_in_Healthcare__Development_and.5.aspx	2011	US	empirical
**15**	Guo, K. L. (2003). An assessment tool for developing healthcare managerial skills and roles. Journal of Healthcare Management / American College of Healthcare Executives, 48(6), 367–376; discussion 376.	2003	USA	empirical
**16**	Hernandez S. R., O'Connor S. J. (2019). The Case for Healthcare Leader Competencies: Exploring the Evidence. International Hospital Federation (IHF). World Hospitals and Health Services Journal, 55(2), 12-15. https://www.ihf-fih.org/download_doc_file.php?doc=67886e31945e37a58b7293be58630d13	2019	US	other – Lit. review
**17**	Howard P. F., Liang Z., Leggat S., Karimi L. (2018). Validation of a management competency assessment tool for health service managers. Journal of Health Organization and Management, 32(1), 113-134. https://www.emerald.com/insight/content/doi/10.1108/JHOM-08-2017-0223/full/html	2018	Australia	empirical
**18**	Jafari M., Nemati A., De Roodenbeke E. (2019). Competencies of Hospital Managers: Iran's Case Study. International Hospital Federation (IHF). World Hospitals and Health Services Journal, 55(2), 21-28. https://www.ihf-fih.org/download_doc_file.php?doc=5e8d76f7c4668ef88b08a31a692ee61c	2019	Iran	empirical
**19**	Kakemam, E., & Dargahi, H. (2019). The Competencies Gap in Hospital Management in Tehran, Iran: A Cross-sectional Survey. JOURNAL OF HEALTH MANAGEMENT, 21(4), 451–464. https://doi.org/10.1177/0972063419884412	2019	Iran	empirical
**20**	Kakemam, E., Liang, Z., Janati, A., Arab-Zozani, M., Mohaghegh, B., & Gholizadeh, M. (2020). Leadership and Management Competencies for Hospital Managers: A Systematic Review and Best-Fit Framework Synthesis. *Journal of Healthcare Leadership*, *12*, 59–68. https://doi.org/10.2147/JHL.S265825	2020	N/A	other – Lit. review
**21**	Kakemam, E., Janati, A., Mohaghegh, B., Gholizadeh, M., & Liang, Z. M. (2021). Developing competent public hospital managers: a qualitative study from Iran. INTERNATIONAL JOURNAL OF WORKPLACE HEALTH MANAGEMENT, 14(2), 149–163. https://doi.org/10.1108/IJWHM-07-2020-0120	2021	Iran	empirical
**22**	Kalhor, R., Tajnesaei, M., Kakemam, E., Keykaleh, M. S., & Kalhor, L. (2016). Perceived hospital managerial competency in Tehran, Iran: is there a difference between public and private hospitals? The Journal of the Egyptian Public Health Association, 91(4), 157–162. https://doi.org/10.1097/01.EPX.0000508180.48823.cd	2016	Iran	empirical
**23**	Khadka, D. K., Gurung, M., & Chaulagain, N. (2014). Managerial competencies–A survey of hospital managers' working in Kathmandu valley, Nepal. Journal of Hospital Administration, 3(1), 62–72.	2014	Nepal	empirical
**24**	Landry A.Y., Stowe M., Haefner J. (2012). Competency assessment and development among health-care leaders: results of a cross-sectional survey. Health Services Management Research, 25(2), 78-86. https://journals.sagepub.com/doi/full/10.1258/hsmr.2012.012012	2012	US	empirical
**25**	Leggat, S. G. (2007). Teaching and learning teamwork: competency requirements for healthcare managers. *The Journal of Health Administration Education*, *24*(2), 135–149.	2007	Australia	empirical

**26**	Lehr, B., Ostermann, H., & Schubert, H. (2011). Competence-based demands made of senior physicians: an empirical study to evaluate leadership competencies. Zeitschrift Fur Evidenz, Fortbildung Und Qualitat Im Gesundheitswesen, 105(10), 723–733. https://doi.org/10.1016/j.zefq.2010.08.006	2011	Germany	empirical
**27**	Liang Z, Howard P, Leggat S. 360° Management Competency Assessment: is our understanding adequate? Asia Pacific Journal of Human Resources (online since 2016,12 February). https://onlinelibrary.wiley.com/doi/10.1111/1744-7941.12108	2016	Australia	empirical
**28**	Liang, Z. and Howard, P.F. (2010), "Competencies required by senior health executives in NSW, 1990-1999", Australian Health Review, Vol. 34 No. 1, pp. 52-58. https://www.publish.csiro.au/AH/AH09571	2010	Australia	empirical
**29**	Liang, Z., Howard, P., & Wollersheim, D. (2017). Assessing the Competence of Evi-dence-Informed Decision-Making Amongst Health Service Managers. Asia Pacific Journal of Health Management, 12(3), 16-23. https://doi.org/10.24083/apjhm.v12i3.53	2017	Australia	empirical
**30**	Liang Z, Blackstock FC, Howard PF, et al. An evidence-based approach to understanding the competency development needs of the health service management workforce in Australia. BMC Health Serv Res. 2018;18(1):976. doi:10.1186/s12913-018-3760-z	2018	Australia	empirical
**31**	Liang, Z., Leggat, S. G., Howard, P. F., & Koh, L. (2013). What makes a hospital manager competent at the middle and senior levels? Australian Health Review, 37(5), 566–573.	2013	Australia	empirical
**32**	Liang, Z. et al. (2018) 'Development and validation of health service management competencies.', Journal of health organization and management, 32(2), pp. 157–175. doi: 10.1108/JHOM-06-2017-0120.	2018	Australia	empirical
**33**	Liang, Z., Howard, P., Wang, J., Xu, M., & Zhao, M. (2020). Developing senior hospital managers: does "one size fit all"? - evidence from the evolving Chinese health system. *BMC Health Services Research*, *20*(1), 281. https://doi.org/10.1186/s12913-020-05116-6	2020	China	empirical
**34**	MacKinnon, N.J., Chow, C., Kennedy, P.L., Persaud, D.D., Metge, C.J. and Sketris, I. (2004), "Management competencies for Canadian health executives: views from the field", Healthcare Management Forum, Vol. 17 No. 4, pp. 15-20. https://journals.sagepub.com/doi/pdf/10.1016/S0840-4704%2810%2960624-2	2004	Canada	empirical
**35**	Mahdavi, A., Ardabili, F. S., Kheirandish, M., Ebrahimpour, H., & Daryani, S. M. (2020). Presenting a Model of Managerial practical wisdom in Hospitals. MANAGEMENT-POLAND, 24(2), 20–48. https://doi.org/10.2478/manment-2019-0045	2020	Iran	empirical
**36**	Malmoon, Z., Tourani, S., Maleki, M., & Jafari, M. (2020). Future competencies for hospital management in developing countries: Systematic review. Medical Journal of the Islamic Republic of Iran, 34, 15. https://doi.org/10.34171/mjiri.34.15	2020	N/A	other – Lit. review
**37**	Martins, J. M., Isouard, G. ., Malik, A. M., & Freshman, B. (2022). Senior Manager Percep-tions of The Human Dimension of Health Ser-vices Management: Australia and Brazil. Asia Pacific Journal of Health Management, 17(1). https://doi.org/10.24083/apjhm.v17i1.929	2022	Australia, Brazil	empirical
**38**	Mehrnoosh, J., Maher, A., & Sheikhi, M. (2020). Transformational Management Properties among Managers of Private and Public Hospitals in Gonbad-e-Kavus City. Journal of Medicine and Life, 13(3), 362–370. https://doi.org/10.25122/jml-2018-0066	2020	Iran	empirical
**39**	Messum, D. G., Wilkes, L. M., Jackson, D., & Peters, K. (2016). Employability Skills in Health Services Management: perceptions of recent graduates. *Asia Pacific Journal of Health Management*, *11*(1), 25-34.	2016	Australia	empirical
**40**	Ogbonnia Godfry O, Chikezie N. managerial competency among hospital managers: does experience on the job matters. J Soc Sci Manag Res. 2018;4(3):88-103.	2018	Nigeria	empirical

**41**	Okonkwo, U., Ekpeyoung, B., Ndep, A., & Nja, G. (2020). Managerial competencies-A survey of healthcare managers in a tertiary hospital in Calabar, South-South Nigeria. Nigerian Journal of Clinical Practice, 23(7), 988–994. https://doi.org/10.4103/njcp.njcp_667_19	2020	Nigeria	empirical
**42**	Patnaik SK, Gupta SK, Kant S, Pillay R.<br>Analyzing Competencies of Indian Health Care Leaders: Way Forward for Next Generation. Int J Res Foundation Hosp<br>Healthc Adm 2017;5(1):47-53. (1):47-53.	2017	India	empirical
**43**	Pillay, R. (2008). The skills gap in hospital management in the South African public health sector. Journal of Public Health Management and Practice: JPHMP, 14(5), E8-14. https://doi.org/10.1097/01.PHH.0000333890.68140.61	2008	South Africa	empirical
**44**	Pillay, R. (2008). Managerial competencies of hospital managers in South Africa: a survey of managers in the public and private sectors. Human Resources for Health, 6, 4. https://doi.org/10.1186/1478-4491-6-4	2008	South Africa	empirical
**45**	Pillay, R. (2008). Defining competencies for hospital management A comparative analysis of the public and private sectors. LEADERSHIP IN HEALTH SERVICES, 21(2), 99–110. https://doi.org/10.1108/17511870810870547	2020	South Africa	empirical
**46**	Pillay, R. (2010) 'The skills gap in hospital management: a comparative analysis of hospital managers in the public and private sectors in South Africa.', Health services management research, 23(1), pp. 30–36. doi: 10.1258/hsmr.2009.009015.	2010	South Africa	empirical
**47**	Ramirez C. L., Ramirez B., Belendez A. H. (2019). Public-Private Partnerships (PPPs) in Healthcare: Gauging Leadership Competen-cies of Hospital Managers. International Hos-pital Federation (IHF). World Hospitals and Health Services Journal, 55(2), 29-33. https://www.ihf-fih.org/download_doc_file.php?doc=0ef175a70aaf679f918eb9a1629a518e	2019	Mexico	empirical
**48**	Selsor, W. (2021). Managerial competencies driving successful change initiatives: A multiple case study of healthcare administrators. In Dissertation Abstracts International: Section B: The Sciences and Engineering (Vol. 82, Issues 4-B). ProQuest Information & Learning.	2021	US	other - Dissertation
**49**	Shewchuk RM, O'Connor SJ, Fine DJ. Building an understanding of the competencies needed for health administration practice. Healthc Manage. 2005; 50(1):32-47. https://pubmed.ncbi.nlm.nih.gov/15729906/	2005	US	empirical
**50**	Shojaei, P., Mokhtari, P. and Rashvand, N. (2011) 'Evaluation of managers competencies of teaching hospitals of Qazvin university of medical science based on 360° degree feed back 2010', World Applied Sciences Journal, 13(1), pp. 60–65.	2011	Iran	empirical
**51**	Stefl, M. E. (2008). Common competencies for all healthcare managers: The healthcare leadership alliance model. *Journal of Healthcare Management*, *53*(6), 360–373. https://doi.org/10.1097/00115514-200811000-00004	2008	US	other - Guidelines
**52**	Toygar, S. A., & Akbulut, Y. (2013). Managerial Skills of Hospital Administrators: Case Study of Turkey. *Journal of Health Management*, *15*(4), 579–594. https://doi.org/10.1177/0972063413516228	2013	Turkey	empirical
**53**	Van Tuong, P., & Thanh, N. D. (2017). A leadership and managerial competency framework for public hospital managers in Vietnam. AIMS Public Health, 4(4), 418.	2017	Vietnam	empirical
**54**	Wallick, W. G., & Stager, K. J. (2002). Healthcare managers' roles, competencies, and outputs in organizational performance improvement / Practitioner response. Journal of Healthcare Management, 47(6), 390–392. https://www.proquest.com/scholarly-journals/healthcare-managers-roles-competencies-outputs/docview/206729167/se-2?accountid=47074	2002	USA	empirical

**55**	Walsh, A. P., Harrington, D., & Hines, P. (2020). Are hospital managers ready for value-based healthcare? A review of the management competence literature. *INTERNATIONAL JOURNAL OF ORGANIZATIONAL ANALYSIS*, *28*(1), 49–65. https://doi.org/10.1108/IJOA-01-2019-1639	2020	N/A	other – Lit. review
**56**	Wongprasit N. The leadership competencies model of private hospital directors in Thailand. HRD J. 2014;4(1):72–85.	2014	Thailand	empirical
**57**	Lockhart W, Backman A, editors. Health care management competencies: identifying the GAPs. Healthcare Management Forum; 2009: Elsevier.	2009	Canada	empirical

Supplementary Table S12. Overview of the included literature review.

No	Abbrev.	Review purpose/objective	Study topic[a]	Type of manager[b]	No. of studies included	**Key findings:** *hospital manager's competencies, skills & behaviors*	Main conclusions
**1**	Calhoun et al. (2002)	*To facilitate discussions associated with competency modeling in health care management education by providing: (1) a summary of recent progress in competency identification for health management; (2) an historical overview on competency-based education and assessment; (3) a glossary of terms frequently used in discussions surrounding competency-based education and training; and (4) an outline of the key challenges and benefits associated with competency modeling.*	T	S, M, N	Not stated	*Goleman Competency Model—(technical, cognitive, and emotional intelligence);* ***Competency Assessment Tool Approach— (****industry knowledge; analytical and conceptual reasoning; and interpersonal and emotional intelligence);* ***Accreditation Council For Graduate Medical Education—(*** *patient care knowledge, practice-based learning and improvement, interpersonal and communication skills, etc.);* ***Competency Clusters for Financial Health Care Roles—(*** *business knowledge, make it happen (e.g., results orientation, etc.), and leading others);* ***Skill Combinations Most Desired by Employers— (****managing self, communicating, managing people and tasks, and mobilizing innovation and change);* ***The Body of Knowledge for Medical Practice Management— (*** *professionalism, leadership, communication skills, organizational and analytical skills, and technical skills);* ***Healthcare Information and Management Systems Society—(*** *General knowledge (e.g., health care, technology, etc.), system knowledge, and administration capabilities);* ***American College of Healthcare Executives— (*** *knowledge of governance and organizational structure, human resources, health care technology, quality and performance improvement, laws and ethics, Management and Business, etc.).*	*The role and responsibility of healthcare educators is to prepare future healthcare managers to cope in an ever-changing environment. It is therefore imperative that the essential management competencies for the future be identified and integrated into and across all training, education, and development curricula for the profession.* ***Defining a broad set of competencies based on the well-documented needs of future healthcare executives will greatly facilitate communication and collaboration across all segments and organizations involved in the training and development of future healthcare leaders.***
**2**	Flaig et al. (2020)	*To understand how individual competencies and organizational outcomes are influenced when hospital managers and leaders*	T, A	S, M	*23*	*Hospital managers and leaders who attended a formal LDP revealed a variety of beneficial outcomes: gained management and leadership knowledge; increased confidence; improved communication skills; improved planning skills; increased self-awareness; teamwork;*	*For hospital managers and leaders who participate in formal LDPs, there can be a variety of positive outcomes. Executives should work to*

		participate in a formal and structured leadership development program (LDP).				*problem-solving skills; ability to encourage others; knowledge of health care systems and processes; positive impact (patient satisfaction, etc.); ability to become a mentor/role model; gained financing/budget skills; improved networking skills; increased approachability; increased understanding of diversity, etc.*	*ensure that the LDP material is achieving the desired beneficial outcome for their organization.*
**3**	Hernandez et al. (2019)	*To explore the relationship between competent management of healthcare organizations and its effect on performance.*	A	S, M, N	Not stated	*Firstly, a relationship between management and leadership competence or behaviors and outcomes at the team or unit level has been documented. Secondly, eight recent research articles have identified the implications of leadership competencies for performance at the organizational level. Lastly, three large studies found a strong and positive link between leadership and management practices and hospital performance.*	*Research shows a positive link between strong health care management and improved performance outcomes in healthcare settings at the individual, team, organizational, and system levels. The findings support the assertion that better management can lead to improved performance in health-care settings.*
**4**	Kakemam et al. (2020)	*To synthesize the evidence related to the leadership and management competencies in healthcare organizations through the best-fit method.*	T	S, M	*12*	*Competencies and behavioural items generated a* ***competency model for hospital managers*** *(applicable in different healthcare contexts). This comprises* ***seven core leadership and management competencies:*** *evidence-informed decision-making; operations, administration, and resource management; knowledge of the healthcare environment and the organization; interpersonal, communication, and relationship management; leading people and organizations; enabling and managing change; and professionalism.*	*The review and mapping of the competencies identified in previous studies against the validated MCAP framework has resulted in the r****ecommendation for an extended leadership and management competency framework*** *for health service managers. It provides guidance for the formulation of training and development directions for the health service management workforce in a different healthcare context.*

**5**	Malmoon et al (2020)	*To determine the future competencies of hospital managers in developing countries.*	T	S, M	*33*	*Core skills (communication skills, conflict and risk management, negotiation skills, change management, time and stress control, personnel management , identifying and solving problems, evidence-based decision making, operational planning, etc.); global knowledge (health economics, cultural management, policy analysis, patient rights, laws, business knowledge, etc.);* ***key eligibility criteria (*** *education, accreditation, accountability, socializations, needs assessment, research, planning and evaluation, etc.); and* ***wide attitude (****emotions management, trust and empathy, political awareness, bioethics, international attitudes, understanding the different kinds of incentives, etc.).*	*It is essential to explain the future competencies of hospital managers to improve efficiency and responsiveness to the changing role of hospitals. In addition to the core competencies that are necessary for management in the present and future, having a broad attitude towards global environmental and knowledge changes and management in the field of information and communication technology will be the most important competencies of a hospital administrator in the future.*
**6**	Walsh (2020)	*To undertake a systematic review of the available literature on managerial competencies in hospitals and consider these in a value-based health-care context.*	T	S, M	*22*	*Leadership (leadership skills and behavior, shaping culture, leading change);* ***Hospital management and health-care environment*** *(hospital organization and health-care system, human resource management, building patient and internal customer relationships);* ***Business skills*** *(evidence-based informed decision-making, operations management, process management and improvement ,risk management, quality and safety, project management, and financial management);* ***Relationship management*** *(communication skills, conflict management);* ***Professional ethics and social responsibility*** *(professionalism, etc.);* ***General management skills*** *(time management, strategic thinking, effective planning, scheduling of tasks and activities).*	*The competency domains and sub-domains identified provide useful guidance for developing management competency models in hospitals operating in a value-based healthcare system, but if used, they would* ***need to be refined further based on the specific requirements of the management level, the management role, and the area of clinical specialization****.*

SUPPLEMENTARY FIGURES

Supplementary Figure S1. The PRISMA of Results.

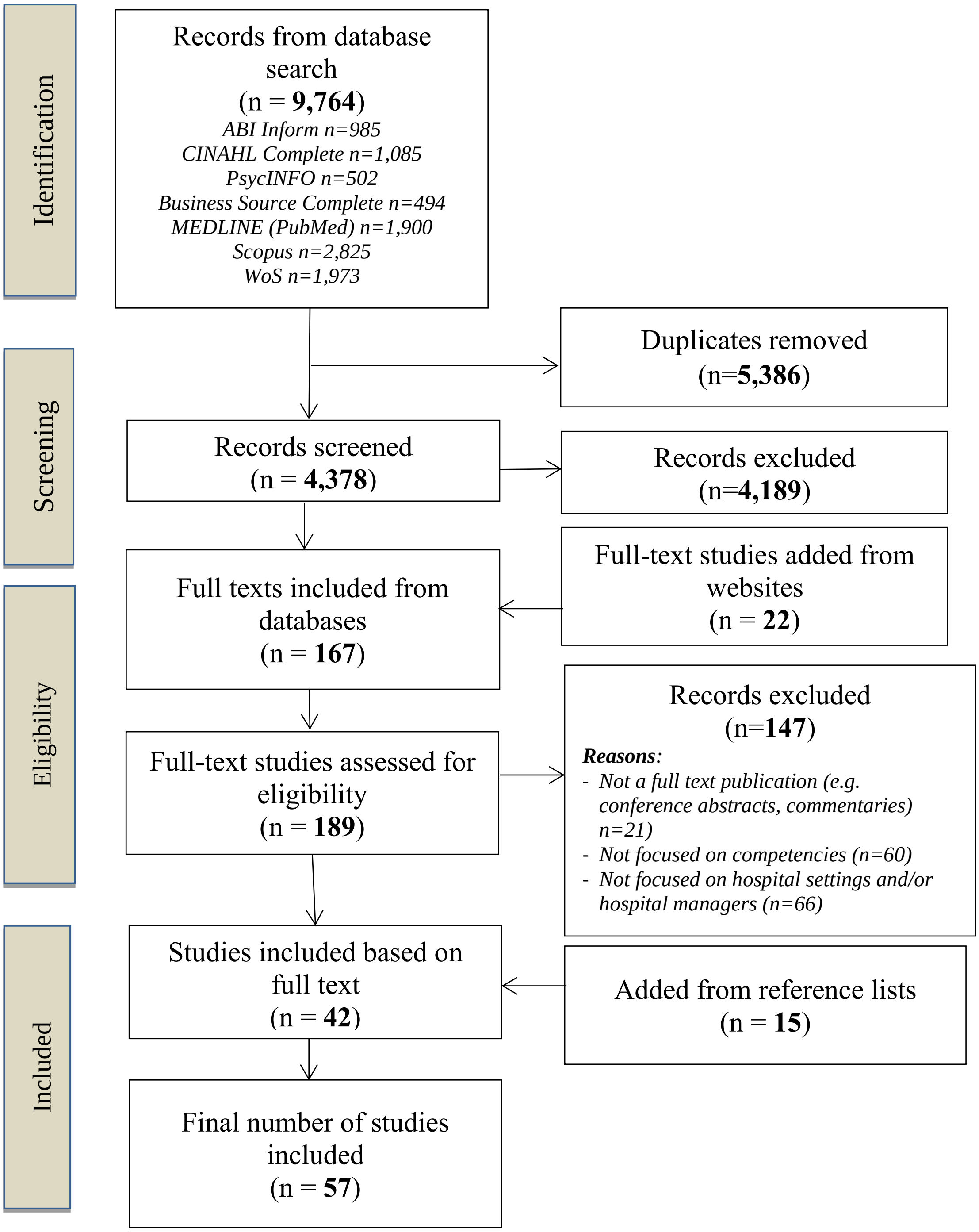

Supplementary Figure S2. Number of empirical studies by region and country

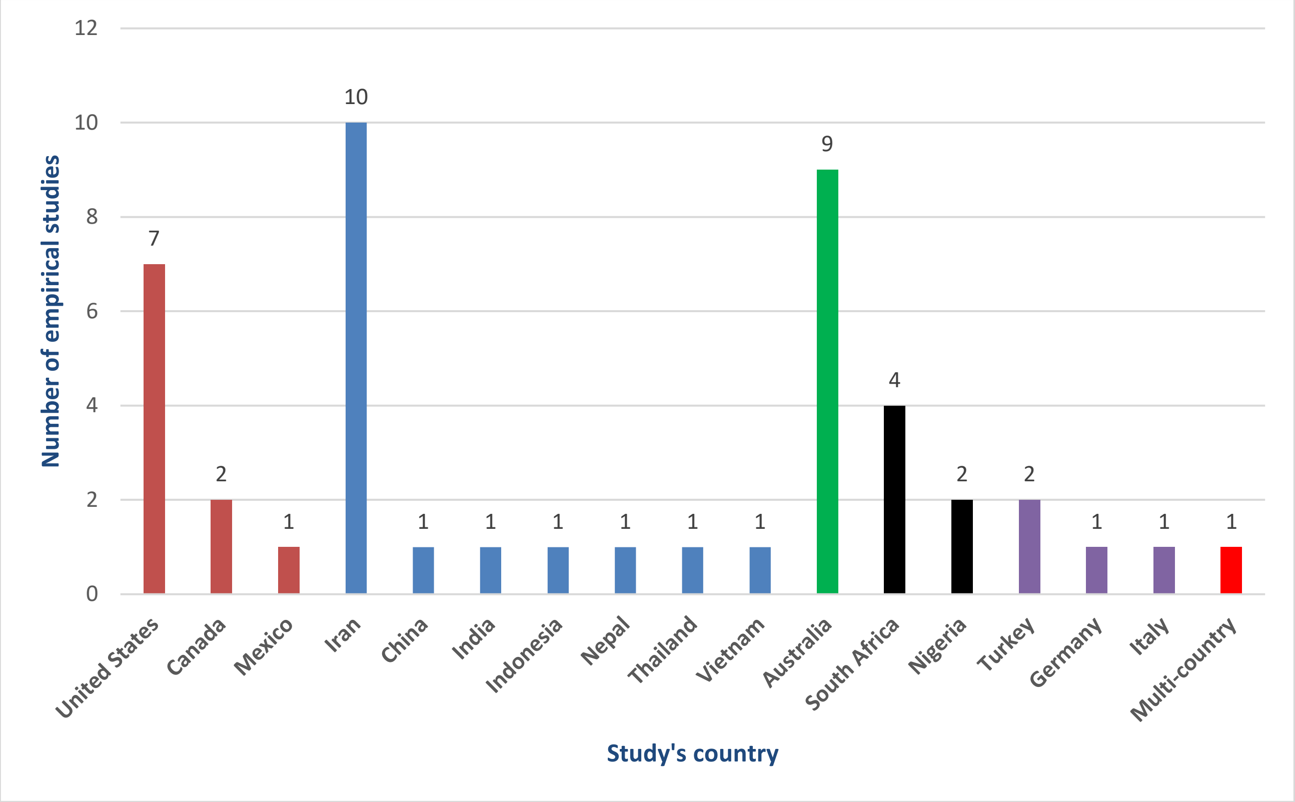

Supplement: Supplementary file 1 [file Data_Sheet_1.pdf]
